# Computational cytometer based on magnetically modulated coherent imaging and deep learning

**DOI:** 10.1038/s41377-019-0203-5

**Published:** 2019-10-02

**Authors:** Yibo Zhang, Mengxing Ouyang, Aniruddha Ray, Tairan Liu, Janay Kong, Bijie Bai, Donghyuk Kim, Alexander Guziak, Yi Luo, Alborz Feizi, Katherine Tsai, Zhuoran Duan, Xuewei Liu, Danny Kim, Chloe Cheung, Sener Yalcin, Hatice Ceylan Koydemir, Omai B. Garner, Dino Di Carlo, Aydogan Ozcan

**Affiliations:** 1 Electrical and Computer Engineering Department, University of California, Los Angeles, CA 90095 USA; 2 Department of Bioengineering, University of California, Los Angeles, CA 90095 USA; 3 California NanoSystems Institute, University of California, Los Angeles, CA 90095 USA; 4 Department of Physics and Astronomy, University of Toledo, Toledo, OH 43606 USA; 5 Department of Physics and Astronomy, University of California, Los Angeles, CA 90095 USA; 6 Yale School of Medicine, New Haven, CT 06510 USA; 7 Department of Biochemistry, University of California, Los Angeles, CA 90095 USA; 8 Department of Pathology and Laboratory Medicine, University of California, Los Angeles, CA 90095 USA; 9 Department of Mechanical and Aerospace Engineering, University of California, Los Angeles, CA 90095 USA; 10 Jonsson Comprehensive Cancer Center, University of California, Los Angeles, CA 90095 USA; 11 Department of Surgery, David Geffen School of Medicine, University of California, Los Angeles, CA 90095 USA

**Keywords:** Biophotonics, Interference microscopy, Imaging and sensing

## Abstract

Detecting rare cells within blood has numerous applications in disease diagnostics. Existing rare cell detection techniques are typically hindered by their high cost and low throughput. Here, we present a computational cytometer based on magnetically modulated lensless speckle imaging, which introduces oscillatory motion to the magnetic-bead-conjugated rare cells of interest through a periodic magnetic force and uses lensless time-resolved holographic speckle imaging to rapidly detect the target cells in three dimensions (3D). In addition to using cell-specific antibodies to magnetically label target cells, detection specificity is further enhanced through a deep-learning-based classifier that is based on a densely connected pseudo-3D convolutional neural network (P3D CNN), which automatically detects rare cells of interest based on their spatio-temporal features under a controlled magnetic force. To demonstrate the performance of this technique, we built a high-throughput, compact and cost-effective prototype for detecting MCF7 cancer cells spiked in whole blood samples. Through serial dilution experiments, we quantified the limit of detection (LoD) as 10 cells per millilitre of whole blood, which could be further improved through multiplexing parallel imaging channels within the same instrument. This compact, cost-effective and high-throughput computational cytometer can potentially be used for rare cell detection and quantification in bodily fluids for a variety of biomedical applications.

## Deep Learning Cytometry: Magnetically modulating rare cells for high-throughput detection

Rare cells of medical significance can be detected in blood by a high-throughput imaging technique that analyzes movements of magnetically-labelled target cells using deep-learning. Analysis of magnetically-modulated light interference using an artificial neural network enhances the ability of the system to specifically and sensitively detect the tagged cells as they respond to a periodically changing magnetic field. Researchers in the USA, led by Aydogan Ozcan at the University of California, Los Angeles, demonstrated the technique using rare cancer cells added to blood. They achieved a detection limit as low as ten cells per milliliter. The optical system uses a holographic process called speckle imaging, which is much more compact and achieves higher throughput than traditional methods. It holds significant potential for the rapid analysis of blood and other bodily fluids to diagnose and monitor disease.

## Introduction

Rare cell detection aims to identify a sufficient number of low-abundance cells within a vast majority of background cells, which typically requires the processing of large volumes of biological sample. The detection and enumeration of these rare cells are vital for disease diagnostics, the evaluation of disease progression and the characterization of immune response^1–3^. For instance, circulating foetal cells present in maternal blood are recognized as a source of foetal genomic DNA, and their isolation is crucial for the implementation of routine prenatal diagnostic testing^4^. As another example, antigen-specific T cells in peripheral blood play a central role in mediating immune response and the formation of immunological memory, which could lead to the prediction of immune protection and diagnosis of immune-related diseases^5^. Circulating endothelial cells with a mature phenotype are increased in patients with certain types of cancer and several pathological conditions, indicating their potential as disease markers^6^. Circulating tumour cells (CTCs) are implicated in various stages of cancer, and have therefore been collected to study their role in the metastatic cascade and to predict patient outcomes from both the disease and treatments received^7^. To highlight yet another example, haematopoietic stem and progenitor cells, which reside predominantly in bone marrow with low numbers, also found in peripheral blood, possess the unique capacity for self-renewal and multilineage differentiation, and their trafficking in blood may be connected to disease processes^8^.

The specific and sensitive detection of these rare cells in human blood and other bodily fluids is therefore of great interest. However, millions of events need to be acquired to obtain a sufficient number of these low-abundance cells (e.g., typically <1000 target cells per millilitre of blood^9^). The direct detection of rare cells from whole blood requires the processing of large amounts of patient sample (e.g., up to a few hundred millilitres^10^), which is both unrealistic and time consuming. To alleviate this issue, highly specific labelling methods are often used before detection for sample purification/enrichment to facilitate rapid detection and processing^5,10^. Among these labelling techniques, the use of colloidal magnetic particles as labelling reagents offers benefits in forming stable suspensions, fast reaction kinetics^10^ and minimum damage to the target cells, with high viability retained^11^.

Motivated by these important needs and the associated challenges, various technologies have been developed and employed for detecting rare cells in blood. Most of these existing detection methods involve three steps: capture, enrichment and detection^12^. The capture and enrichment steps use a number of methods, such as barcoded particles^13^, magnetic beads^14^, micro-machines^15^, microfluidic chips^16^ and density gradient centrifugation^12,17^. Following the enrichment step, these rare cells can be detected via commonly used techniques, such as immunofluorescence^18,19^, electrical impedance^20^ and Raman scattering^21^ measurements, among others. Notably, commercial products for rare cell detection, such as the CellSearch system^22^, which automates magnetic labelling, isolation, fluorescence labelling and automated counting, are generally high cost, limiting their adoption worldwide^12^. Therefore, cost-effective, reliable and high-throughput rare cell detection techniques are urgently needed to improve the early diagnosis of diseases, including cancer, so that earlier treatments can be carried out, helping us to improve patient outcomes while also reducing healthcare costs^23,24^.

**Fig. 1:**
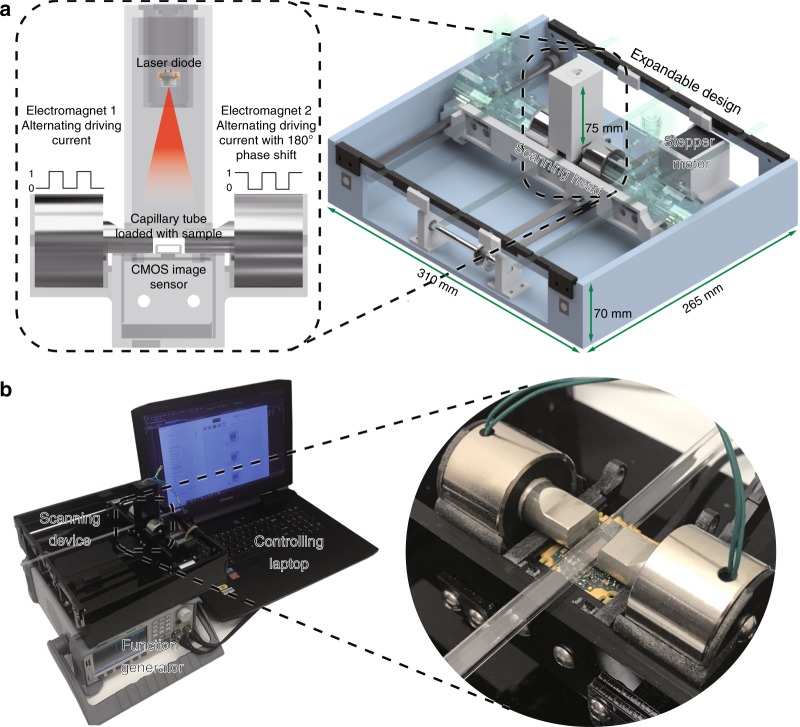
Schematics and photos of the computational cytometer. **a** A magnetically modulated lensless imaging module (inset) that includes a lensless holographic microscope and two electromagnets driven by two alternating currents with opposite phase. The fluid sample that contains magnetic-bead-conjugated cells of interest is loaded into a capillary tube. The imaging module is mounted to a linear motion stage to scan along the sample tube to record holographic images of each section of the tube. **b** A laptop computer is used to control the device and acquire data. A function generator and a power supply, together with custom-designed circuitry, are used to provide the power and driving current for the linear motion stage and electromagnets

The recent advances in machine learning and, specifically, deep learning have pushed the frontiers of biomedical imaging and image analysis^25–38^, enabling rapid and accurate pathogen detection^39–42^ and computer-assisted diagnostic methods^43–47^. Powered by deep learning, we demonstrate here that speckle imaging using lensless chip-scale microscopy can be employed for the specific and sensitive detection of rare cells in blood with low cost and high throughput. This novel cell detection and cytometry technique are based on magnetically modulated lensless speckle imaging, which specifically labels rare cells of interest using magnetic particles attached to surface markers of interest and generates periodic and well-controlled motion on target cells by alternating the external magnetic field applied to a large sample volume. The holographic diffraction and the resulting speckle patterns of the moving cells are then captured using a compact and cost-effective on-chip lensless imager (Fig. 1), and are computationally analysed by a deep-learning-based algorithm to rapidly detect and accurately identify the rare cells of interest in a high-throughput manner based on their unique spatio-temporal features. Although previous work has employed the idea of using magnetic modulation for enhancing fluorescence detection^48,49^, our work is the first of its kind for combining magnetic modulation, lensless imaging and deep learning to create a unique cytometer that does not require additional labelling (e.g., fluorescence) or custom-designed molecular probes.

**Fig. 2:**
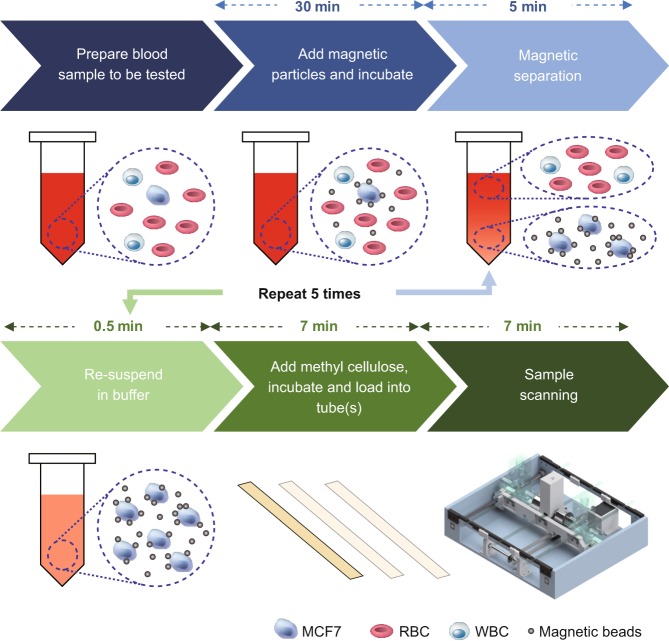
Sample preparation and imaging procedures. The sample preparation time before scanning is ~1 h, with the first 30 min dedicated to passive incubation, which does not require supervision

As shown in Fig. 1, we built a portable prototype of this computational cytometer for rare cell detection. Our magnetically modulated speckle imaging module includes a lensless in-line holographic microscope^41,50–57^ and two oppositely positioned electromagnets (Fig. 1a, b inset). The lensless microscope contains a laser diode (650 nm wavelength) to illuminate the sample from ~5–10 cm above, and a complementary metal–oxide–semiconductor (CMOS) image sensor is placed ~1 mm below the sample for acquisition of a high-frame-rate video to monitor the spatiotemporal evolution of the sample containing the target cells of interest. Because the light-source-to-sample distance is much greater than the sample-to-image-sensor distance, the optical design has a unit magnification, and the field of view (FOV) of a single image is equal to the active area of the image sensor (which can be 10–30 mm^2^ using the standard CMOS imagers employed in digital cameras and mobile phones). To increase the screening throughput, target cells are enriched using magnetic separation and loaded inside a capillary tube for imaging (Figs. 1, 2). Magnetic enrichment alone leads to a background of unlabelled cells, bead clusters or weakly labelled cells that are also captured, such that further discrimination of the target cells within this background information is needed to accurately identify and count the rare cells. The imaging module is mounted onto a custom-made linear translation stage, and is translated along the direction of the sample tube to capture a holographic video for each section of the sample tube. During the imaging at each section, the electromagnets are supplied with alternating current with a 180° phase difference to exert an alternating pulling force to the magnetic-bead-conjugated cells in the sample, which causes them to oscillate at the same frequency as the driving current. Extension rods made of permalloy were designed and utilized to enhance the magnetic force at the sample location by ~40-fold (see the Methods section and Fig. S1). The holographic diffraction patterns that are cast by the magnetically modulated target cells are captured using the image sensor and transferred to a laptop computer. A computational motion analysis (CMA) algorithm^41^ and a densely connected pseudo-3D convolutional neural network structure (P3D CNN)^58^ then analyse the holographic image sequence that contains the 3D dynamic information from the oscillating cells, which allows rapid and specific detection of the target cells.

The current prototype (Fig. 1) screens ~0.942 mL of fluid sample, corresponding to ~1.177 mL of whole-blood sample before enrichment, in ~7 min (Fig. 2), while costing only ~$750 for the raw materials (excluding the function generator, power supply and laptop computer) and weighing ~2.1 kg. The platform with a single imaging channel can be expanded to parallel imaging channels by mounting several imaging modules onto the same linear stage, as shown in Fig. 1a (semi-translucent illustrations).

The performance of our technique was tested by detecting a model rare cell system of spiked MCF7 cancer cells in human blood. We demonstrate that our technique has a limit of detection (LoD) of 10 cells per millilitre of whole blood using only a single-imaging channel. Because the current LoD is mainly limited by the screening volume, we expect that the LoD can be further improved by including additional parallel imaging channels and increasing the sample volume that is screened.

## Results

### Characterization of the oscillation of bead–cell conjugates under alternating magnetic force

Our detection technique capitalizes on the periodic oscillatory motion of the target cells of interest, with a large number of labelling magnetic particles, to specifically detect them with high throughput. We designed a magnetic actuator to exert periodic and alternating magnetic force on the magnetic particles bound to these cells of interest (Fig. 1). To exert sufficient magnetic force on each labelled cell, we designed and machined extension rods that were made with magnetically soft permalloy, which were attached to the electromagnets to enhance the magnetic force at the sample location by ~40-fold with minimal magnetic hysteresis (see the Methods section and Fig. S1).

**Fig. 3:**
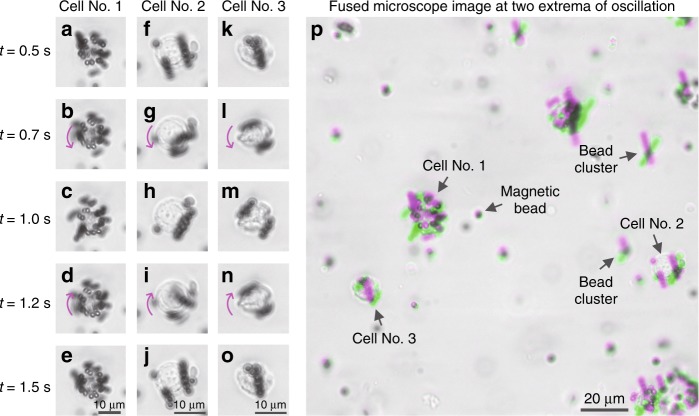
Dynabead-conjugated MCF7 cells demonstrate periodic rotational motion under an alternating magnetic force field. Images were acquired using a 40 × 0.6NA benchtop microscope. **a**–**o** Snapshots of three Dynabead-conjugated MCF7 cells at different time points within a period of oscillation (period = 1 s). **p** Images taken at the two extrema of the oscillation (*t* = 0.5 s and *t* = 1.0 s) were fused together to demonstrate the movement, where the grey regions in the fused image represent the consistency between the two images and the magenta/green colours represent the differences of the two images. Magenta represents the first image (*t* = 0.5 s), and green represents the second image (*t* = 1.0 s)

The movement of MCF7 cells conjugated with Dynabeads was recorded by mounting the magnetic actuator and the labelled cells onto a 40 × 0.6NA benchtop microscope (see Fig. 3). The sample preparation procedure is depicted in Fig. 2, where the Dynabead-conjugated cells were suspended in a methyl cellulose solution (a viscous fluid) and were subjected to alternating magnetic fields with a period of 1 s and a square-wave driving current. As shown in Fig. 3a–o and Video S1, due to the high viscosity of the methyl cellulose solution, the labelled cells mainly demonstrated 3D rotational motion. Typically, the motion of a labelled cell starts at the beginning of a cycle of the magnetic field (e.g., *t* = 0.5 s), approaching a steady state (e.g., *t* = 1.0 s) before the magnetic field switches its direction and the cell rotates in the reverse direction (e.g., between *t* = 1.0 s and *t* = 1.5 s). The two extreme positions of the rotational motion are demonstrated in Fig. 3p by overlaying the images captured at *t* = 0.5 s and *t* = 1.0 s using magenta and green, respectively.

Various unbound magnetic beads and bead clusters are also observed within the sample (Fig. 3p reports some examples, marked with text and arrows), which also oscillate at the same frequency as that of the bead-conjugated target cells. If not handled properly, these might form a major cause of false positives. However, the spatio-temporal dynamics of bead-conjugated cells significantly differ from those of unbound beads and bead clusters (see the following subsections and the Methods section). For a given amount of magnetic driving force, the bead-conjugated cells are subjected to more inertia and viscous drag, which is manifested by a slower response to the magnetic field, i.e., a slower rotational motion. In addition, magnetic beads typically form chains when they cluster under an external magnetic field, and these chains exhibit a swinging motion under the alternating magnetic field. This contrasts with the 3D rotational motion, i.e., the “rolling” motion associated with the bead-conjugated cells (see Video S2 for comparison). These intricate spatio-temporal dynamic features, in addition to morphological differences, are utilized by a subsequent classification step (based on a deep neural network) to achieve higher accuracy and eliminate false positive detections, as will be detailed in the following subsections and the Methods section.

### Cell detection and classification using CMA and deep learning

**Fig. 4:**
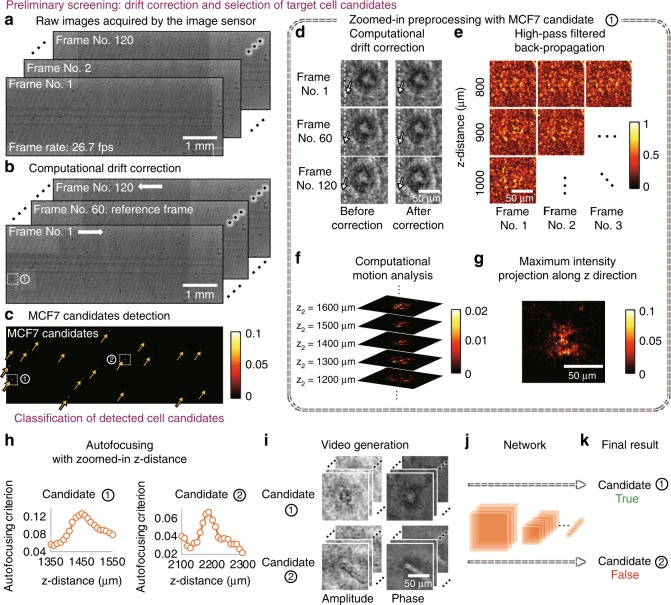
Computational detection of rare cells. **a**–**c** Preliminary screening of the whole FOV to detect candidates for target cells (MCF7). At each scanning position, 120 frames of raw holograms were taken at 26.7 frames per second. Computational drift correction was applied to mitigate the horizontal shift caused by the fluid drift, where the vertical movement caused by the magnetic field was kept unmodified. The lateral position of each MCF7 candidate was located by CMA, maximum intensity projection and threshold-based detection. **d**–**g** Zoomed-in preliminary processing for the example region labelled ① in **b**, **c**. **h**–**k** Classification process for the two cell candidates labelled ① and ② in **c**. The axial location for each cell candidate was determined by autofocusing. A video was formed for each cell candidate by propagating each frame to the in-focus plane. The classification was performed by a densely connected P3D convolutional neural network, as detailed in the Methods section

The sample, which contains the periodically oscillating target cells and other types of unwanted background particles, is illuminated with coherent light. The interference pattern recorded by the CMOS image sensor represents an in-line hologram of the target cells, which is partially obscured by the random speckle noise resulting from the background particles, including other unlabelled cells, cell debris and unbound magnetic particles. Recorded at 26.7 frames per second using the CMOS image sensor, these patterns exhibit spatio-temporal variations that are partially due to the controlled cell motion. This phenomenon is exploited for the rapid detection of magnetic-bead-conjugated rare cells from a highly complex and noisy background. Figure 4a–g shows the detailed computational steps for the preliminary screening of cell candidates from a raw holographic image sequence. First, a computational drift correction step mitigates the overall drift of the sample between frames. Then, a high-pass filtered back-propagation step using the angular spectrum method^59^ calculates the holographic images at different axial distances within the 3D sample. A CMA step analyses the differences among the frames to enhance the 3D contrast for periodically moving objects that oscillate at the driving frequency and employs time averaging to suppress the random speckle noise caused by background particles. This is then followed by a maximum intensity projection and threshold-based detection to locate potential cell candidates.

The cell candidates that are detected in this preliminary screening step contain a large number of false positives, which mainly result from unbound magnetic beads that form clusters under the external magnetic field. Therefore, we employ another classification step (Fig. 4h–k) to improve the specificity of our final detection. For this classification step, we choose to use a densely connected P3D CNN structure to classify the holographic videos to exploit the spatial and temporal information encoded in the captured image sequence. The densely connected P3D CNN structure is modified based on a recently proposed CNN structure^58^ by adding dense connections^60^. Compared with other machine-learning techniques, the use of a deep neural network for video classification is typically more powerful, and the network can be retrained to classify other types of cells or objects of interest^58,61^.

An autofocusing step^62,63^ is applied to each candidate object to create an in-focus amplitude and phase video, which is then classified (as positive/negative) by a densely connected P3D CNN. These classification results are used to generate the final rare cell detection decisions and cell concentration measurements. The CNN was trained and validated with manually labelled video clips generated from ten samples that were used solely for creating the training/validation data sets. This training needs to be performed only once for a given type of cell-bead conjugate (for details, refer to the Methods section).

### Evaluation of system performance

To quantify the LoD of our platform for detecting MCF7 cells in human blood, we spiked cultured MCF7 cells in whole blood at various concentrations and used our technique to detect the spiked MCF7 cells. Using spiked samples instead of clinical samples provides a well-defined system to characterize and quantify the capabilities of our platform, which is an important step before moving to clinical samples in the future.

In each experiment, 4 mL of MCF7-spiked whole human blood at the desired concentration was prepared. Then, the procedure in Fig. 2 was followed to perform magnetic separation and embed the recovered cells in the viscous methyl cellulose medium, resulting in ~3.2 mL of final sample volume. This prepared sample was then loaded into a disposable capillary tube to be screened by our computational cytometer. Because the capillary tube length is designed to be longer than the range of the motion of the linear stage and because the capillary tube was wider than the width of the CMOS sensor, the actual imaged volume per test (within the sample tube) is ~0.942 mL, which corresponds to ~1.177 mL of the blood sample before the enrichment process.

**Fig. 5:**
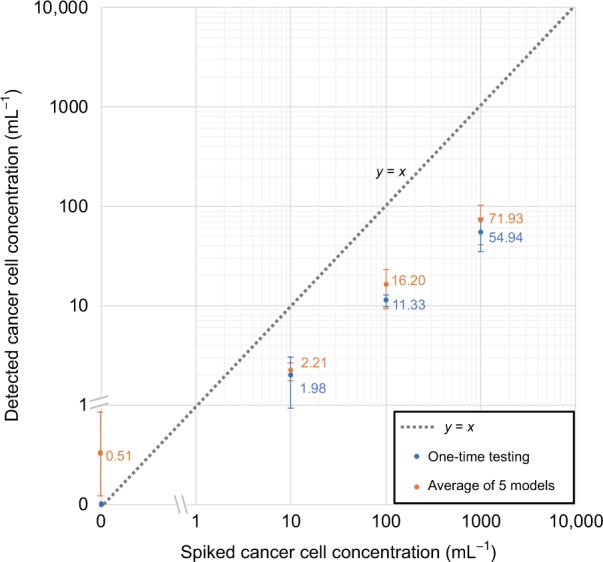
Quantification of the LoD of our computational cytometer based on magnetically modulated lensless speckle imaging for the detection of MCF7 cells in whole blood. The axes are a hybrid of logarithmic and linear scales to permit 0 cell/mL to be shown in the same plot. The blue data points represent one-time testing results of a single trained P3D CNN. The error bars represent the respective standard deviation of the three repeated tests at each spiked target cell concentration. The orange data points represent the averaged testing results using five P3D CNNs that were individually trained on a different subset of data. The error bars represent the standard deviation resulting from the detections of the five individual networks; for each trained network, three detected concentrations are averaged at each spiked concentration

MCF7 concentrations of 0 mL^−1^ (negative control), 10 mL^−1^, 100 mL^−1^ and 1000 mL^−1^ were tested, where three samples for each concentration were prepared and independently measured. Figure 5 shows the results of the blind testing of our technique using serial dilution experiments. The blue data points correspond to a one-time testing result, where the error bars correspond to the standard deviations of the three detected concentrations at each spiked concentration. Without the detection of any false positives in the negative control samples, our technique was able to consistently detect MCF7 cells from 10 mL^−1^ samples, measuring a target cell concentration of 1.98 ± 1.06 mL^−1^. At this low concentration (10 cells/mL), the detection rate was ~20%. The experimentally measured detection rate dropped to ~5% at a higher concentration of 1000 cells/mL.

Because the training of the deep neural network inherently includes randomness, we further evaluated the repeatability of our network training process. For this, we randomly and equally divided our training data into five subsets, and then we trained five individual networks by assigning one different subset as the validation data set and the combination of the remaining four subsets as the training data set. Each of the five networks was blindly tested to generate the serial dilution results. The mean and standard deviation of the detected concentrations resulting from the five networks are shown in Fig. 5 (orange data points; for each trained network, three detected concentrations are averaged at each spiked concentration). Overall, good consistency between the different network results is observed.

The underdetection behaviour of our system is due to a combination of both systematic errors and random factors. A major reason for underdetection is the tuning of the classification network. In the preliminary screening step, because there are typically a large number of false-positive detections and a low number of true-positive detections (since the target cells are quite rare), our classifier must be tuned to have an extremely low false-positive rate (FPR) to have a low LoD. To satisfy this, we applied a widely adopted method for tuning our classifier^64^, where we selected a decision threshold based on the training/validation data set, which leads to a zero FPR (see the Methods section for details). However, an inevitable side effect of reducing the FPR is a reduction in the true positive rate (TPR). Based on the validation results, when a decision threshold of 0.999999 was used, the TPR dropped to 10.5%. This explains a major portion of the reduced detection rate that we observed in the serial dilution tests (Fig. 5). Another systematic error that contributes to the underdetection is the imperfect recovery rate of MCF7 cells during the enrichment. We experimentally quantified the recovery rate of MCF7 cells using Dynabeads to be ~85% (Table S1).

The remainder of the underdetection and fluctuations in the detection rate at different concentrations may be associated with various other factors, e.g., sample handling errors (especially at low cell concentrations), clustering of the target cells, and non-uniform labelling of cells with magnetic beads. In fact, MCF7 cells are known to form clusters and have thus been extensively used for preparing in vitro tumour models^65,66^. In an experiment where we spiked MCF7 cells at a concentration of 1.1 × 10^5^/mL (Table S1), we observed that ~50% of the MCF7 cells formed clusters after enrichment. However, the amount of clustering is expected to be lower at decreased MCF7 concentrations, which partially explains our reduced detection efficiency at higher cell concentrations. This clustering of cells not only reduces the overall number of target entities but may also exhibit changes in their oscillation patterns and may be misclassified by our classifier.

## Discussion

The presented computational cytometry technique may be applied for the detection of various types of rare cells in blood or other bodily fluids using appropriately selected ligand-coated magnetic beads. There are several advantages of our magnetically modulated speckle imaging technique. The first important advantage is its ability to detect target rare cells without any additional modification, such as labelling with fluorescent or radioactive compounds, unlike the vast majority of the existing techniques. The same magnetic beads that are used for capturing and isolating target cells from whole blood are also used for periodic cell modulation and specific detection within a dense background. False positives are mitigated by identifying the controlled spatio-temporal patterns associated with the labelled target cells through a trained deep neural network.

Compared with existing approaches, our technique also has the advantages of a relatively low LoD, rapid detection and low cost, which makes it suitable for the sensitive detection of rare cells in resource-limited settings. For example, fluorescence imaging and Raman microscopy have been widely used to detect rare cells and have been shown to have very low LoDs (e.g., ~1 cell/mL)^12,67,68^, but they are typically limited by a high system cost and complexity. To address this issue, a low-cost fluorescence system for detecting rare cells was introduced by Balsam et al.^69^, which detects fluorescently labelled cells flowing in a fluidic channel using laser excitation and a low-cost camera for imaging. They demonstrated a LoD comparable to ours (~10 mL^−1^) for SYTO-9-labelled THP-1 monocytes in whole blood. However, the use of fluorescence labelling can suffer from the drawback of photobleaching. As another notable example for sensitive and cost-effective rare cell detection, Issadore et al. proposed using Hall sensors to detect magnetic-bead-labelled target cells in a microfluidic channel and demonstrated a high sensitivity in detecting CTCs^70^. However, their technique requires a relatively long detection time (2.5 h) and a strong expression of biomarkers in target cells. Other rare cell detection technologies, such as chemiluminescence detection based on aptamer-specific cell capture^71^ and DNA-oriented shaping of cell features^72^, have also been reported, but their capabilities were demonstrated using only cell mixtures in a buffer solution with limited throughput, i.e., 3 µL^71^ or 500 µL^72^ cell solution per batch.

In our approach, while deep-learning-based classification is instrumental to achieving high detection accuracy, it needs to be retrained on different types of cells, which requires collecting and labelling a large amount of data for each new type of target cell. This is a disadvantage of our approach; however, preparing the training data and manually labelling the target cells is not prohibitively time consuming, and it needs to be performed only once, during the training phase. For example, when we prepared the training/validation data for MCF7 cells, we used 10 experiments to create a manually labelled library containing 17,447 videos of candidate cells (including positives and negatives). The manual labelling process took ~10 h. These procedures only need to be performed once for a given type of target cell. Compared with using fluorescence labelling, which requires additional experimental steps and reagents each time, we believe that this one-time cost of preparing training data for the deep neural network presents advantages.

Another limitation of our method is that it can detect only positive cells, which are labelled with magnetic beads; negative cells that are not labelled are not counted. In addition, in this proof-of-concept study, we only demonstrated our detection technique on a single type of target cell. However, a future direction would be to explore the feasibility of multiplexed labelling for different types of target cells. One possibility for multiplexing is to use magnetic particles of different sizes (e.g., varying from ~100 nm to 10 µm), shapes and iron content, where each type of magnetic particle is coated with the corresponding antibody that is specific to a different type of cell. In this approach, different cell-bead conjugates would have distinct dynamics when they are subjected to a varying magnetic force field, which would lead to different patterns of oscillation that can be specifically detected^48,49^. The cell-bead conjugates may also exhibit different responses to magnetic modulation when the frequency is varied. These spatio-temporal and morphological signatures may be classified by an appropriately designed and trained deep-learning-based classifier. Therefore, any type of rare cell that can be specifically identified/isolated using antibodies or any targeting moieties can potentially be targeted using our presented system.

The spatial resolution and the quality of the images captured using our system are degraded by the random speckle noise generated by background objects, which limits our ability to perform further morphological analysis based on reconstructed images. However, at different frames of a video that is captured with our system, since the target objects of interest (i.e., the bead-labelled MCF7 cells) are modulated with a unique spatio-temporal pattern, thereby exposing different perspectives of the cells (as demonstrated in Video S2), a robust distinction of the target cells from the background is achieved using our deep-learning-based video classifier.

The entire prototype of our computational cytometer shown in Fig. 1b (excluding the function generator, power supply and laptop computer) has a raw material cost of ~$750. This cost can be significantly reduced under large volume manufacturing, and currently it is mainly attributed to the image sensor and frame grabber (~$550), the permalloy rod (~$70) and the electromagnets (~$40), with the other components being much more inexpensive. In future versions of this instrument, the power supply and function generator can be replaced with cost-effective integrated circuit chips. For example, the power supply can be replaced with a 20 V power adapter (e.g., TR9KZ900T00-IMR6B, GlobTek, Inc., Northvale, NJ, USA) and a step-down converter (e.g., LTC3630EMSE#PBF, Analog Devices, Norwood, MA, USA) to supply 20 V and 12 V for the electromagnets and the stepper motor, respectively; the function generator can be replaced with an oscillator circuit built from a timer integrated circuit (e.g., NE555DR, Texas Instruments, Dallas, TX, USA). The total cost of these components would be <$25. Furthermore, the device can be easily scaled up to include two or more parallel imaging channels to achieve a higher sample throughput, which is proportionate with the number of imaging channels.

## Methods

### Cell preparation

MCF7 cell lines were purchased from ATCC (Manassas, Virginia, USA). Cells were plated with 10 mL of growth media in a T75 flask (Corning Inc., New York, USA) at a concentration of 1 × 10^5^ cells/mL. The growth media was composed of Dulbecco’s Modified Eagle Medium (DMEM, Gibco®, Life Technologies, Carlsbad, California, USA) supplemented with 10% (v/v) foetal bovine serum (FBS, Gibco®, Life Technologies, Carlsbad, California, USA) and 1% penicillin–streptomycin (Sigma-Aldrich Co., St. Louis, Missouri, USA). Cells were grown in a humidified incubator at 37 °C in a 5% CO_2_ environment. Cells were harvested by treating them with 0.25% trypsin-EDTA (Gibco®, Life Technologies, Carlsbad, California, USA) for 3 min 2 to 3 days after seeding, depending on confluency. Then, the cells were pelleted by centrifuging for 3 min at 1200 RPM and resuspended in the growth media to a final concentration of 1 × 10^6^ cells/mL.

### Sample preparation

*Rare cell dilution*: The MCF7 cells were serially diluted in Dulbecco’s phosphate-buffered saline (DPBS, Sigma-Aldrich Co., St. Louis, Missouri, USA) at different concentrations (2 × 10^4^ cells/mL, 2 × 10^3^ cells/mL, and 2 × 10^2^ cells/mL). The dilution of MCF7 cells in whole blood was prepared by mixing the cell solution with whole blood at a ratio of 1:19 (v/v). Most of the experiments were performed by mixing 200 μL of cell solution with 3.8 mL of whole blood. Healthy human whole blood (from anonymous and existing samples) was obtained from the UCLA Blood and Platelet Center.

*Bead washing*: CELLection Epithelial Enrich Dynabeads (Invitrogen, Carlsbad, California, USA) were first resuspended in DPBS and vortexed for 30 s. A magnet (DX08B-N52, K&J Magnetics, Inc., Pipersville, Pennsylvania, USA) was then used to separate the Dynabeads, and the supernatant was discarded. This process was repeated three times, and the Dynabeads were resuspended in DPBS at the initial volume.

*Rare cell separation*: The washed Dynabeads were then added to the MCF7-spiked whole blood sample at a concentration of 2.5 μL beads per 1.0 mL of blood sample. The mixture was incubated for 30 min with gentle tilting and rotation. A magnet was placed under the vial for 5 min, and the supernatant was discarded after that. To this solution, we added 1 mL of cold DPBS buffer and mixed it gently by tilting from side to side. This magnetic separation procedure was repeated five times. After the final step, the sample was resuspended in 0.7 mL of DPBS and gently mixed with 2.5 mL of 400 cP methyl cellulose solution (Sigma-Aldrich Co., St. Louis, Missouri, USA) using a pipette. The sample was incubated for 5 min to reduce the number of bubbles before it was loaded into a glass capillary tube (Part # BRT 2-4-50; cross-section inner dimension of 2 mm × 4 mm; $11.80 per foot; Friedrich & Dimmock, Inc., Millville, New Jersey, USA). The ends of the capillary tube were sealed with parafilm before the tube was mounted onto our computational cytometer for imaging and cell screening.

### Design of the computational cytometer based on magnetically modulated lensless speckle imaging

As shown in Fig. 1, our device hardware consists of an imaging module and a linear translation module. The imaging module, i.e., the scanning head in Fig. 1, contains a laser diode (650 nm wavelength, AML-N056-650001-01, Arima Lasers Corp., Taoyuan, Taiwan, China) for illumination, which has an output power of ~1 mW. The sample is loaded inside a capillary tube with a rectangular cross-section, which is placed ~7.6 cm below the light source. A CMOS image sensor (acA3800-14um, Basler, Ahrensburg, Germany) with a pixel size of 1.67 μm, which is placed below the glass tube with a narrow gap (~1 mm), is used to capture the holographic speckle patterns generated by the liquid sample. To induce oscillatory motion to the labelled cells in the sample, two electromagnets (Part #XRN-XP30 × 22, Xuan Rui Ning Co., Ltd., Leqing, Zhejiang Province, China) with custom-machined permalloy extensions are placed on either side of the glass tube. An alternating driving current (square wave) is supplied to either of the electromagnets, with a 180° phase shift between them, which creates alternative pulling force to the magnetic particles within the sample. The low level of the driving current is 0, and the high level of the driving current is ~500 mA. The frequency is 1 Hz, which was experimentally optimized to maximize the signal corresponding to the magnetic-bead-conjugated cancer cells.

The linear translation stage is custom-built using off-the-shelf components. A bipolar stepper motor (No. 324, Adafruit Industries LLC., New York, USA) with two timing pulleys and a timing belt is used to provide mechanical actuation, and the imaging module is guided by a pair of linear motion sliders and linear motion shafts on either side of the scanning head. 3D-printed plastic is used to construct the housing for the scanning head, and laser-cut acrylic is used to create the outer shell of the device.

### Image acquisition

After the sample is loaded into the capillary tube and placed onto our computational cytometer, the image acquisition procedure begins. The linear translation stage moves the scanning head to a series of discrete positions along the glass tube. At each position, the stage stops allowing the CMOS image sensor to capture a sequence of 120 holograms at a frame rate of 26.7 fps before moving onto the next position. The image data are saved to a solid-state drive (SSD) for storage and further processing.

Because the FOV corresponding to the edges (i.e., top and bottom rows) of the image sensor is subject to a highly unbalanced magnetic force field due to the closeness to one of the electromagnets, only the central 1374 rows of the image sensor’s pixels are used to capture the image sequence, where the magnetic force from the two electromagnets are relatively balanced.

Because the CMOS image sensor temperature quickly rises when it is turned on, it tends to cause undesired flow inside the glass tube due to convection. Therefore, a scanning pattern is engineered to reduce the local heating of the sample: if we denote 1, 2, …, 32 as the indices of the spatially adjacent scanning positions, the scanning pattern follows 1, 9, 17, 25, 2, 10, 18, 26, …. This scanning pattern ensures that a given part of the sample cools down before the scanning head moves back to its neighbourhood. The power to the image sensor is also cut-off during the transition between the two successive scanning positions, which is implemented by inserting a MOSFET-based switch into the power line of the USB cable.

### Computational detection and localization of cell candidates and deep-learning-based classification

The image processing procedure (Fig. 4) can be divided into two parts: (1) a preliminary screening step, which applies computational drift correction and MCF7 candidate detection to the entire FOV to locate target cell candidates in 2D, and (2) a classification step, which refocuses the holographic image sequence to each individual MCF7 candidate in its local area, generates an in-focus amplitude and phase video for each candidate, and classifies the corresponding video with a trained deep neural network. This procedure is further detailed below.

#### 1. Preliminary screening

##### Computational drift correction

The sample fluid in the glass capillary tube often drifts slowly throughout the duration of the image acquisition, which is due to, e.g., the imperfect sealing at the ends of the tube and the convection due to the heat of the image sensor. Because the detection and classification of the target cells are largely based on their periodic motion, the drifting problem should be corrected. Since our sample is embedded within a viscous methyl cellulose, minimal turbulent flow is observed and the drifting motion within our imaged FOV is almost purely translational. We used a phase correlation method^73^ to estimate the relative translation between each frame in the sequence with respect to a reference frame (chosen to be the middle frame in the holographic image sequence) and used 2D bilinear interpolation to remove the drift between frames. As shown in Fig. S2, this drift correction step successfully removed many false positive detections in the CMA step due to the background drift.

##### Detection of target cell candidates

The detection of the target cell candidates plays a key role in automatically analysing the sample, as it greatly narrows down the search space for the rare cells of interest and allows the subsequent deep-learning-based classification to be applied to a limited number of holographic videos. In the preliminary screening stage, the lateral locations of the MCF7 candidates are detected. Each frame of the raw hologram sequence is propagated to various axial distances throughout the sample volume using a high-pass-filtered angular spectrum propagation kernel, which can be written as:

1 $${\mathbf{B}}_{i}\left( {z_j} \right) = {{HP}}\left[ {\mathcal{P}}\left( {{\mathbf{A}}}_{i},z_{j} \right) \right]$$

where *HP*(·) denotes the high-pass filter (see Supplementary Information for details), ${\mathcal{P}}$ (·) denotes angular spectrum propagation^59^, **A**_*i*_ denotes the *i*th frame of the raw hologram sequence after the drift correction, and *z*_*j*_ denotes the *j*th propagation (axial) distance. The selected propagation distances ranged from 800 μm to 5000 μm with a step size of 100 μm to ensure coverage of all possible MCF7 candidates within the sample tube. A zoomed-in image of **B**_*i*_(*z*_*j*_) corresponding to an example region is shown in Fig. 4e.

Next, for every given propagation distance, a CMA algorithm is applied to reveal the oscillatory motion of the target cells within the sample, which focuses on periodic changes in the recorded frames:

2 $${\mathbf{C}}\left( {z_j} \right) = \frac{1}{{N_{\mathrm{F}} - N}}\mathop {\sum}\limits_{i = 1}^{N_{\mathrm{F}} - N} {\left( {\frac{1}{2}\left| {{\mathbf{B}}_{i}\left( {z_j} \right) - {\mathbf{B}}_{i + N/2}\left( {z_j} \right)} \right| +\, \frac{1}{2}\left| {{\mathbf{B}}_{i + N/2}\left( {z_j} \right) - {\mathbf{B}}_{i + N}\left( {z_j} \right)} \right| - \left| {{\mathbf{B}}_{i}\left( {z_j} \right) - {\mathbf{B}}_{i + N}\left( {z_j} \right)} \right|} \right)}$$

where **C**(*z*) and **B**(*z*) are shorthand notations for **C**(*x*, *y*; *z*) and **B**(*x*, *y*; *z*), respectively, *N*_F_ is the total number of recorded frames (in our case, *N*_F_ = 120), and *N* is chosen such that the time difference between the *i*th frame and the (*i* + *N*)th frame is equal to the period of the alternating magnetic field. Therefore, the first two terms inside the summation in Eq. (2) represent half-period movements at the *j*th propagation distance, and the last term represents the whole-period movement. Ideally, for objects that oscillate periodically with the alternating magnetic force field, the first two terms should be relatively large, and the last term should be relatively small. For randomly moving objects, the three terms in the summation approximately cancel each other out. As a result, **C**(*x*, *y*; *z*) is a 3D contrast map that has high values corresponding to the locations of periodic motion that matches the frequency of the external magnetic field. An example of **C** is shown in Fig. 4f.

To simplify segmentation, a maximum intensity projection along the axial direction (i.e., *z*) is applied to flatten the 3D image stack into a 2D image, which can be written as:

3 $${\mathbf{D}}\left( {x,y} \right) = \mathop {{{\mathrm{max}}}}\limits_z \left[ {{\mathbf{C}}\left( {x,y;z_1} \right),{\mathbf{C}}\left( {x,y;z_2} \right),...,{\mathbf{C}}\left( {x,y;z_{N_{\mathrm{H}}}} \right)} \right]$$

where *x* and *y* are the lateral indices and *N*_H_ is the total number of axial positions (in our case, *N*_H_ = 43). An example of **D** is shown in Fig. 4c, with a zoomed-in image shown in Fig. 4g. Thresholding-based segmentation was applied to the calculated 2D image **D**, and the resulting centroids are used as the lateral positions of the MCF7 candidates.

#### 2. Classification

##### Autofocusing and video generation

After the preliminary screening, which identifies the lateral centroids of potential target cell candidates, the subsequent processing is applied to each MCF7 candidate only within their local area. Autofocusing^62,63^ was first performed to locate the MCF7 candidate in the axial direction. Because **C**(*x*, *y*; *z*_*j*_) should have a higher value when approaching the in-focus position of each MCF7 candidate, the approximate axial position was obtained by maximizing (as a function of *z*_*j*_) the sum of the pixel values of **C**(*x*, *y*; *z*_*j*_) (*j* *=* 1, 2, …, *N*_H_) in a local neighbourhood around each individual MCF7 candidate. We chose to use a local neighbourhood size of 40 × 40 pixels (i.e., 66.8 μm × 66.8 μm). This process can be written as follows:

4 $$\hat z_k = \mathop {{{\mathrm{arg}}\,{\mathrm{max}}}}\limits_{z_j = 1,2,...,N_H} \mathop {\sum}\limits_{x,y = - 19}^{20} {{\mathbf{C}}\left( {x_k + x,y_k + y;z_j} \right)}$$

where $\widehat z_k$ is the resulting in-focus position for the *k*th potential target cell candidate and *x*_*k*_ and *y*_*k*_ are the lateral centroid coordinates of the *k*th potential target cell candidate.

The same criterion to find the focus plane can be applied again with finer axial resolution to obtain a more accurate estimation of the axial distance for each MCF7 candidate. We used a step size of 10 μm in this refined autofocusing step. Two examples of this process are shown in Fig. 4h. Alternatively, the Tamura coefficient^62,63^ could also be used as the autofocusing criterion to determine the in-focus plane.

Finally, the in-focus amplitude and phase video corresponding to each MCF7 candidate was generated by digitally propagating every frame of the drift-corrected hologram sequence to the candidate’s in-focus plane. The final video has 120 frames at 26.67 fps with both the amplitude and phase channels, and each frame has a size of 64 × 64 pixels (pixel size = 1.67 μm). Two examples corresponding to two cell candidates are shown in Fig. 4i.

##### Target cell detection using densely connected P3D CNN

**Fig. 6:**
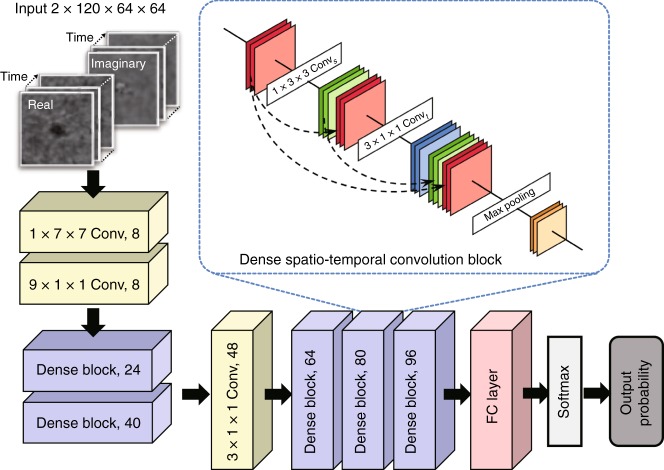
Structure of the densely connected P3D CNN. The network consists of convolutional layers, a series of dense blocks, a fully connected layer and a softmax layer. As shown in the inset, each dense spatio-temporal convolution block was constructed by introducing skip connections between the input and output of the convolutional layers in the channel dimension, where red represents the input of the dense block, green and blue represent the output of the spatial and temporal convolutional layers, respectively, and yellow represents the output of the entire block

Each video of the MCF7 candidate was fed into a classification neural network (Fig. 6), which outputs the probability of having an MCF7 cell in the corresponding video (Fig. 4j, k). We designed a novel structure for the classification neural network, named densely connected P3D CNN, which is inspired by the Pseudo-3D Residual Network^58^ and the Densely Connected Convolutional Network^60^. The original P3D CNN^58^ used a mixture of three different designs of the P3D blocks to gain structural diversity, which resulted in a better performance. In this work, we introduced a densely connected structure to the P3D CNN structure by adding dense (skip) connections inside the spatio-temporal convolution block (dashed black arrows in Fig. 6 inset) to unify the three different P3D blocks. This allowed a simpler network design that was easier to implement for our task.

The detailed structure of the densely connected P3D CNN is shown in Fig. 6. The network contains five densely connected spatio-temporal convolutional blocks. As shown in the inset of Fig. 6, each block consists of a 1 × 3 × 3 spatial convolutional layer (Conv_*s*_), a 3 × 1 × 1 temporal convolutional layer (Conv_*t*_), followed by a max pooling layer (Max). Each spatial (or temporal) convolutional layer is a composition of three consecutive operations: batch normalization, a rectified linear unit (ReLU) and a spatial (or temporal) convolution (with stride = 1 and output channel number equal to the growth rate *k* *=* 8). In each block, we introduced skip connections between the input and output of the Conv_*s*_ layer as well as the Conv_*t*_ layer by concatenating (⊕) the input and the output in the channel dimensions. For a given input tensor *m*_*p*_, the densely connected spatio-temporal convolutional block maps it to the output tensor *m*_*p+1*_, which is given by:

5 $$m_{p + 1} = {\mathrm{Max}}\left[ {{\mathrm{Conv}}_t\left( {{\mathrm{Conv}}_s(m_p) \oplus m_p} \right) \oplus \left( {{\mathrm{Conv}}_s(m_p) \oplus m_p} \right)} \right]$$

For example, consider an input video with a size of *c* × *t* × *h* × *w*, where *c*, *t*, *h* and *w* denote the number of channels, number of frames (time), height and width of each frame (space), respectively. Here, *c* = 2, *t* = 120, and *h* = *w* = 64. We first pass the video through a 1 × 7 × 7 spatial convolutional layer (stride = 2) and a 9 × 1 × 1 temporal convolution layer (stride = 3) sequentially. The output channel numbers of the layers are included in Fig. 6 in each box. Then, the data go through five dense blocks, where between the 2nd and 3rd dense blocks, we add an additional 3 × 1 × 1 (stride = 1) convolutional filter with no padding to ensure that the time and space dimensions are equal. A fully connected (FC) layer with a 0.5 dropout rate and a softmax layer are introduced, which output the class probability (target rare cell or not) for the corresponding input video. Finally, a decision threshold is applied to the class probability output to generate the final positive/negative classification, where the decision threshold is tuned based on the training/validation data to reduce the FPR (detailed in the next sub-section “Network training and validation”).

##### Network training and validation

We performed ten experiments (i.e., ten samples) to create the training/validation data sets for our classifier and then used the trained classifier to perform blind testing on additional serial dilution experimental data (Fig. 5), which had no overlap with the training/validation data. Among the ten experiments for constructing the training/validation data set, five were negative controls, and the other 5 were spiked whole-blood samples at an MCF7 concentration of 10^3^ mL^−1^. When manually labelling the video clips to create the training/validation data set, we noticed that some videos were difficult to label, where the annotators could not make a confident distinction. Therefore, to ensure an optimal labelling accuracy, our negative training data came from only the five negative control experiments, where all the candidate videos from those experiments were used to construct the negative data set. The positive training data were manually labelled by two human annotators using five experiments spiked at 10^3^ mL^−1^, where only the video clips that were labelled as positive with high confidence by both annotators were selected to enter the positive training data set, while all the others were discarded.

Next, the training/validation data sets were randomly partitioned into a training set and a validation set with no overlap between the two. The training set contained 1713 positive videos and 11324 negative videos. The validation set contained 788 positive videos and 3622 negative videos. The training data set was further augmented by randomly mirroring and rotating the frames by 90°, 180° and 270°. The convolutional layer weights were initialized using a truncated normal distribution, while the weights for the FC layer were initialized to zero. Trainable parameters were optimized using an adaptive moment estimation (Adam) optimizer with a learning rate of 10^−4^, and a batch size of 240. The network converged after ~800–1000 epochs. The network structure and hyperparameters were first optimized to achieve high sensitivity and specificity for the validation set. At a default decision threshold of 0.5, a sensitivity and specificity of 78.4% and 99.4%, respectively, were achieved for the validation set; a sensitivity and specificity of 77.3% and 99.5%, respectively, were achieved for the training set. After this initial step, because our rare cell detection application requires the classifier to have a very low FPR, we further tuned the decision threshold of our classifier to avoid false positives. For this, the training and validation data sets were combined to increase the total number of examples, and we gradually increased the decision threshold (for positive classification) from 0.5 while monitoring the FPR for the combined training/validation data set. We found that a decision threshold of 0.99999 was able to eliminate all false-positive detections in the combined training/validation data set. We further raised the decision threshold to 0.999999 to account for potential overfitting of the network to the training/validation data and further reduced the risk of false-positive detections.

At a decision threshold of 0.999999, as expected, the TPR dropped down to 10.5% (refer to Fig. S3, which reports the receiver operating characteristic (ROC) curve based on the validation data set, with an area under the curve of 0.9678). This low TPR results in underdetection of the target cells, as also evident in our serial dilution results (Fig. 5). The selection of the decision threshold is dependent on the specific application of interest and should be tuned based on the expected abundance of target cells and the desired LoD. For the application considered in this work, because the expected number of target cells at the lowest concentration (i.e., 10 mL^−1^) is extremely low, the decision threshold was tuned to a high level to suppress false positives, which in turn resulted in a very low TPR. However, for less demanding cell detection or cytometry applications where the desired LoD is not as stringent, the decision threshold may be relaxed to a lower level, which also allows the TPR to be higher.

##### Computation time

Using our current computer code, which is not optimized, it takes ~80 s to preprocess the data within one FOV (corresponding to a volume of 14.7 mm^2^ × 2 mm) for extracting the MCF7 cell candidates, corresponding to the preliminary screening step in Fig. 4. For each detected cell candidate, it takes ~5.5 s to generate the input video for network classification. The network inference time for each input video is <0.01 s. Based on these numbers, if there are, e.g., ~1500 cell candidates per experiment, the total processing time using the current computer code would be ~3.0 h. However, we should note that the data-processing time depends on various factors, including the computer hardware configuration, the cell concentration in the sample, the programming language and whether the code is optimized for the hardware. In our work, although we used relatively high-performance hardware (an Intel Core i7 CPU, 64 GB of RAM and an Nvidia GeForce GTX 1080Ti GPU) and used some of the GPU functions provided by MATLAB (MathWorks, Natick, MA, USA), we did not extensively optimize our code for improved speed. A careful optimization of the GPU code should bring a significant speedup in our computation time.

### COMSOL simulation of the magnetic force field generated by the electromagnet and the permalloy extension

Because of space constraints, the electromagnet could not be placed sufficiently close to the imaging area, which caused the magnetic force to be low. We used a custom-machined extension rod made of permalloy^74^ (relative permeability *μ*_r_ ~ 100,000) to relay the force field and enhance the relative magnetic force on target cells by ~40 times. To simulate the magnetic force field distribution near an electromagnet with and without the permalloy extension, a finite element method (FEM) simulation was conducted using COMSOL Multiphysics (version 5.3, COMSOL AB, Stockholm, Sweden). A 3D model was developed using the magnetic field interface provided in the COMSOL AC/DC physics package. A stationary study was constructed based on the geometry of a commercially available electromagnet, where the core was modelled with a silicon steel cylinder (radius = 3 mm, height = 10 mm), and the coil was modelled with a surface current of 10 A/m on the side of the core running in the azimuthal direction. The permalloy extension was modelled using Permendur. A thick layer of air was added as a coaxial cylinder with a radius of 10 mm and a height of 30 mm. The magnetic flux density inside the simulation space was simulated using the magnetic field module. Then, a coefficient form PDE module in the mathematics library was used to derive the relative magnetic force field. The magnetic force that is received by superparamagnetic beads is given by:

6 $${\mathbf{F}} = \frac{{V\chi }}{{\mu _0}}\left( {{\mathbf{B}} \cdot \nabla } \right){\mathbf{B}}$$

where *V* is the volume of the magnetic particle, *χ* is the magnetic susceptibility, *μ*_0_ is the magnetic permeability in a vacuum and **B** is the magnetic flux density.

Our simulation results are shown in Fig. S1. The results in Fig. S1b indicate that the relative magnetic force rapidly reduces as a function of the distance from the electromagnet. However, by using a permalloy extension, the relative magnetic force at the sample location is enhanced by ~40 times.

## Supplementary information

Supplementary Information

Supplementary Video 1

Supplementary Video 2
