# Supplementary material for: Computational cytometer based on magnetically modulated coherent imaging and deep learning
Source: Light Sci Appl. 2019 Oct 2;8:91. doi: 10.1038/s41377-019-0203-5 (PMC6804677; doi:10.1038/s41377-019-0203-5)
Supplement: Supplementary file 1 — Supplementary Information [file 41377_2019_203_MOESM1_ESM.pdf]

Supplementary Information for

Computational cytometer based on magnetically modulated coherent imaging and deep learning

Yibo Zhang[1,2,3†], Mengxing Ouyang[2†], Aniruddha Ray[1,2,3,4†], Tairan Liu[1,2,3†], Janay Kong[2], Bijie Bai[1,2,3], Donghyuk Kim[2], Alexander Guziak[5], Yi Luo[1,2,3], Alborz Feizi[1,2,3,6], Katherine Tsai[7], Zhuoran Duan[1], Xuewei Liu[1], Danny Kim[2], Chloe Cheung[2], Sener Yalcin[1], Hatice Ceylan Koydemir[1,2,3], Omai B. Garner[8], Dino Di Carlo[2,3,9,10], and Aydogan Ozcan[1,2,3,11,*]

[1]Electrical and Computer Engineering Department, University of California, Los Angeles, CA 90095, USA
[2]Department of Bioengineering, University of California, Los Angeles, CA 90095, USA
[3]California NanoSystems Institute, University of California, Los Angeles, CA 90095, USA
[4]Department of Physics and Astronomy, University of Toledo, Toledo, Ohio 43606, USA
[5]Department of Physics and Astronomy, University of California, Los Angeles, CA 90095, USA
[6]Yale School of Medicine, New Haven, CT 06510, USA
[7]Department of Biochemistry, University of California, Los Angeles, CA 90095, USA
[8]Department of Pathology and Laboratory Medicine, University of California, Los Angeles, CA 90095, USA
[9]Department of Mechanical and Aerospace Engineering, University of California, Los Angeles, CA 90095, USA
[10]Jonsson Comprehensive Cancer Center, University of California, Los Angeles, CA 90095, USA
[11]Department of Surgery, David Geffen School of Medicine, University of California, Los Angeles, CA 90095, USA

*Correspondence: ozcan@ucla.edu
[†]Equally contributing authors

Supplementary Methods

High-pass filtered back-propagation using the angular spectrum method

The recorded holographic speckle images were back-propagated to different axial distances (i.e., z-distances) using the angular spectrum method[1,2] with a high-pass filtered transfer function.[3] Because the approximate size of the target cells of interest is known a priori, a high-pass filter was factored into the propagation transfer function in the spatial frequency domain, which was useful for suppressing noise and unwanted artifacts.

The free-space propagation transfer function is given by:

$$H\left(f_x, f_y; z\right) = \begin{cases} \exp\left[j\frac{2\pi z}{\lambda}\sqrt{1-\left(\lambda f_x\right)^2-\left(\lambda f_y\right)^2}\right], & \sqrt{f_x^2+f_y^2} \le \frac{1}{\lambda} \\ 0, & \text{others} \end{cases} \tag{1}$$

where z is the distance to propagate, λ is the wavelength of light, and f_x and f_y are spatial frequency coordinates. The high-pass filtered transfer function is modified based on $H\,(f_x, f_y; z)$ as:

$$\tilde{H}\left(f_x, f_y; z\right) = H\left(f_x, f_y; z\right) \cdot \min\left\{G_1\left(f_x, f_y\right), G_2\left(f_x, f_y\right)\right\} \tag{2}$$

where G_1 and G_2 are the high-pass filters in the spatial frequency domain, given by

$$G_1\left(f_x, f_y\right) = 1 - \exp\left[-\frac{1}{2}\sigma_1^2\left(f_x^2 + f_y^2\right)\right] \tag{3}$$

and

$$G_2\left(f_x, f_y\right) = 1 - \exp\left[-\frac{1}{2}\sigma_2^2 f_y^2\right] \tag{4}$$

where σ_1 = 50 µm and σ_2 = 117 µm. G_1 was used mainly to suppress the low-frequency interference patterns caused by the various interfaces in the light path, and G_2 was used mainly to suppress the unwanted diffraction patterns due to the grooves in the capillary tubes, which is a manufacturing artifact.[3]

Supplementary Tables

Table S1. Concentrations of different types of cells and particles in the sample before and after the magnetic enrichment. MCF7 cells were spiked into a whole blood sample at a concentration of 1.1×10^5 mL^{-1}, and enrichment was performed following the procedure reported in Fig. 2. After the enrichment, the sample was loaded into a counting chamber and imaged by a 20× 0.45NA benchtop microscope, and the particles/cells were manually counted. In order to directly compare it with the concentration before the enrichment step, the concentration after the enrichment is normalized by a volume factor (i.e., the ratio between the volume before the enrichment and the volume after the enrichment). As reported in our table, there is over three orders of magnitude decrease in the concentration of blood cells, while the recovery rate for the target MCF7 cells is ~85%. The concentrations of unbound beads and bead clusters reported here only provide an estimate, because bead clusters are also dynamically formed during the experiments, when there is an external magnetic field applied.

	Concentration (mL^{-1})	
	Before enrichment	After enrichment
Total labeled MCF7 cells	1.1×10^5	9.4×10^4
Non-clustering labeled MCF7 cells	-	4.7×10^4
Labeled MCF7 cell clusters	-	1.7×10^4
Blood cells	$\sim5\times10^9$ (estimated based on the average healthy human blood cell concentration)	1.6×10^6
Magnetic beads	-	1.3×10^6
Bead clusters	-	1.1×10^5

Supplementary Figures

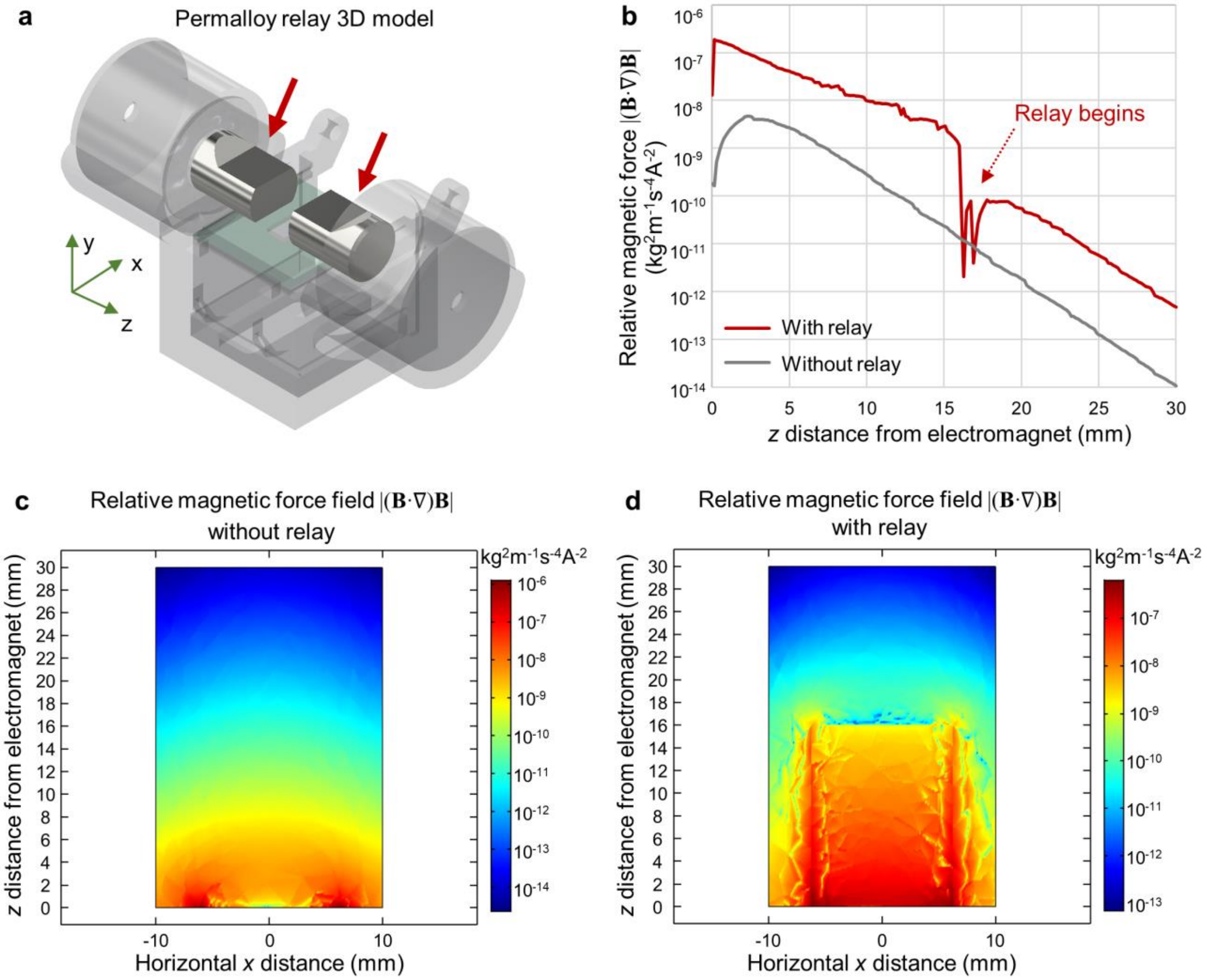

Figure S1. COMSOL simulation of magnetic force field generated by electromagnet with permalloy relay. a 3D schematic of the permalloy relays relative to the electromagnets. **b-d** Simulation of the relative (unitless) magnitude of the magnetic force field that is generated by a single electromagnet with (**c**) or without (**d**) permalloy relay, as a function of the spatial position. The relay significantly increases the magnetic force field for a given axial distance from the electromagnet.

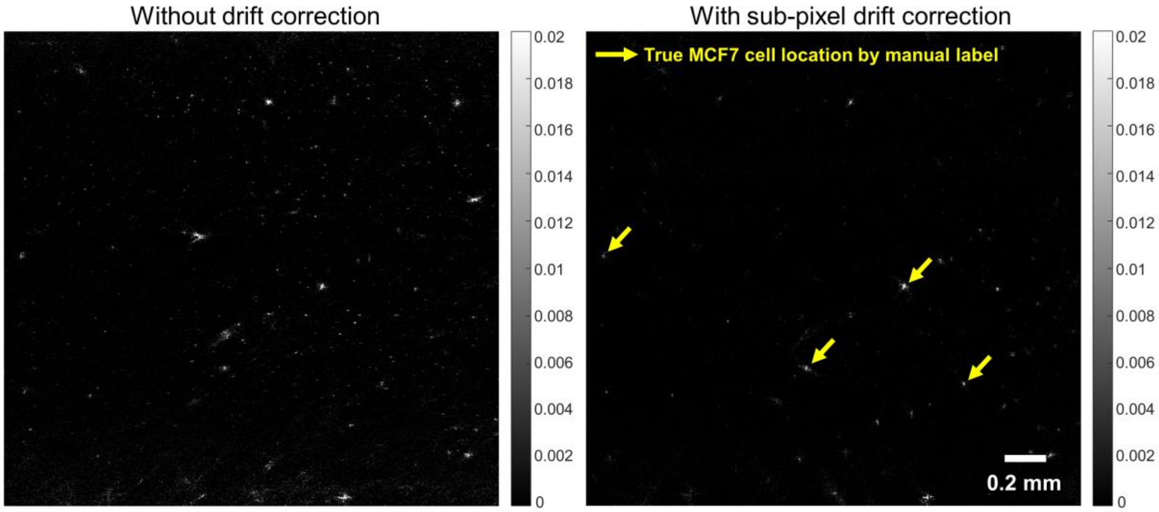

Figure S2. Effect of using computational drift correction to remove false positives. Without drift correction, due to the drifting of the medium, particles that do not oscillate in response to the changing magnetic force field may generate contrast in the 2D contrast map, which reduces the effectiveness of the computational motion analysis. The sub-pixel drift correction step removes most of the “false positive” contrast.

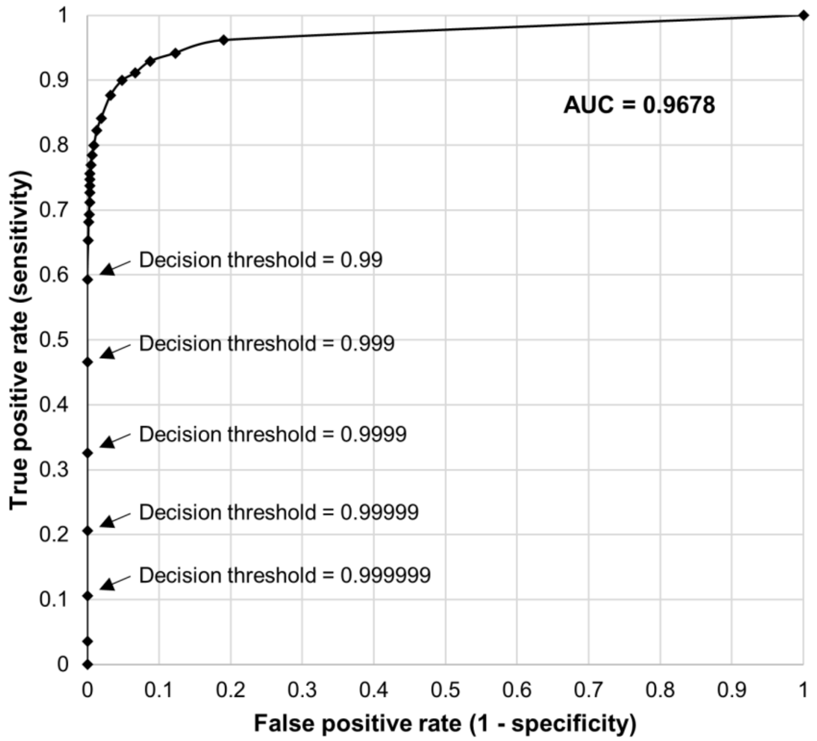

Figure S3. Receiver operating characteristic (ROC) curve of the trained P3D CNN classifier. The curve is generated by varying the decision threshold for positive classification using the validation dataset. The area under the curve (AUC) is 0.9678. The blind serial dilution testing experiments (Fig. 5) are not used to calculate the AUC, because for those tests the ground truth concentrations are based on the dilution factor used during the spiking experiments. Manual labeling was not performed for the serial dilution testing data.

Supplementary Videos

Video S1: Motion of magnetic bead-conjugated MCF7 cells and unbound beads under alternating external magnetic field, captured by a 40× 0.6NA benchtop microscope.

Video S2: Amplitude and phase videos of three magnetic bead-conjugated MCF7 cells and three magnetic bead clusters that were reconstructed using the lensless on-chip imaging platform, which demonstrate their unique spatio-temporal characteristics. Each video is played twice.

Supplementary References

1. Goodman, J. W. *Introduction to Fourier optics*. (Roberts & Co, 2005).
2. Matsushima, K. & Shimobaba, T. Band-limited angular spectrum method for numerical simulation of free-space propagation in far and near fields. *Opt. Express* **17**, 19662–19673 (2009).
3. Zhang, Y. *et al.* Motility-based label-free detection of parasites in bodily fluids using holographic speckle analysis and deep learning. *Light Sci. Appl.* **7**, (2018).
